# Evolving Surgical Timing for Bilateral Hip Arthroscopy in Femoroacetabular Impingement: A Comparison of Clinical Outcomes Between Simultaneous and Staged Approaches

**DOI:** 10.7759/cureus.101292

**Published:** 2026-01-11

**Authors:** Marco Minelli, Marco Rosolani, Vincenzo Longobardi, Alessio D'addona, Antonio Orgiani, Berardo Di Matteo, Federico Della Rocca

**Affiliations:** 1 Department of Biomedical Sciences, Humanitas University, Milan, ITA; 2 Department of Hip and Knee Prosthetic and Minimally Invasive Orthopedic Surgery, Istituto di Ricovero e Cura a Carattere Scientifico (IRCCS) Humanitas Research Hospital, Milan, ITA

**Keywords:** arthroscopy, bilateral procedures, femoroacetabular impingement, hip, single-stage

## Abstract

Background and aim

Femoroacetabular impingement (FAI) commonly presents with radiographic signs in both hips, and many patients report symptoms on both sides. Hip arthroscopy is the preferred surgical treatment for symptomatic FAI. The optimal approach with the lowest procedural risk between simultaneous and staged bilateral arthroscopy remains debated in the literature. The objective of this single-center study was to compare clinical outcomes, complication rates, length of hospital stay, return to sport, and patient satisfaction among patients undergoing single-stage, early-stage, and two-stage bilateral hip arthroscopy for femoroacetabular impingement.

Materials and methods

This monocentric retrospective study evaluated patients undergoing bilateral hip arthroscopy for symptomatic FAI between 2017 and 2023, all performed by a single surgeon. Patients were divided into the following three groups: two-stage (TS; ≥6 weeks between procedures), early-stage (ES; second procedure the day after the first), and single-stage (SS; simultaneous) groups. The group assignment was based on shared decision-making, and propensity score matching was used to balance for age, gender, and BMI. Hip Outcome Score - Activities of Daily Life (HOS-ADL), Hip Outcome Score - Sports Subscale (HOS-Sport), International Hip Outcome Tool-12 (iHOT-12), and visual analog scale (VAS) were collected preoperatively and at two years postoperatively. Major complications, return to sport, and patient satisfaction were also recorded.

Results

The study included 60 TS, 30 ES, and 30 SS patients. Mean cumulative surgical times did not significantly differ between groups. No major complications, revisions, or conversions to total hip replacement were recorded at the two-year follow-up. The total length of hospital stay was significantly shorter in the SS group compared to the TS and ES groups. All clinical outcome scores improved significantly at follow-up. No significant differences were found in HOS, HOS-Sport, iHOT-12, VAS, return to sport, or satisfaction rates between the three groups.

Conclusion

There is no inferiority in clinical outcomes when using a simultaneous approach compared to more staged approaches. The single-stage group had a significantly shorter mean total length of hospital stay compared to the staged groups. The single-stage approach is a viable solution for selected patients and experienced surgical teams, allowing for outcomes that are not inferior to the early-stage or two-stage approaches.

## Introduction

The prevalence of radiographic signs of femoroacetabular impingement (FAI) in asymptomatic populations has been reported to be as much as 29% [1-3]. Among patients suffering from unilateral FAI symptoms, radiographic evidence of pathology on the contralateral side is present in up to 78% of individuals [4]. Whenever there is radiographic evidence of bilateral FAI, up to 52% of patients present with symptoms in both hips [5]. Hip arthroscopy has emerged as the preferred surgical procedure for managing symptomatic FAI, offering favorable outcomes in terms of pain relief and return to activity [6,7]. Bilateral hip arthroscopy can be performed as a single-stage simultaneous procedure or as a two-stage procedure, where each hip is treated in separate surgeries over a defined interval [8,9]. While single-stage surgery may reduce total rehabilitation time and healthcare costs, risk considerations of simultaneous procedures include increased traction and anesthesia time, which could increase the potential risk of neurological and soft-tissue complications, particularly for less experienced surgeons [8-12]. Conversely, two-stage procedures may allow for more controlled recovery and individualized rehabilitation of each hip, but at the cost of two different surgical procedures, two anesthesias, prolonged total recovery time, and increased healthcare utilization [8-12]. Previous studies have reported favorable outcomes for both simultaneous and staged bilateral arthroscopies; however, direct comparisons remain limited [8,9]. In this context, an early-stage approach, where the second hip is treated the day after the first, was introduced to manage technical challenges and reduce the risk of complications. Initially, only staged (two-stage and early-stage) procedures were performed at our center due to surgical experience and institutional protocols. As surgeon confidence and intraoperative protocols improved, simultaneous single-stage procedures were introduced.

## Materials and methods

This monocentric retrospective study evaluated clinical outcomes of a consecutive series of patients undergoing bilateral hip arthroscopy for symptomatic femoroacetabular impingement between 2017 and 2023 at a single tertiary referral center. The present study was approved by the Independent Ethics Committee of Istituto di Ricovero e Cura a Carattere Scientifico (IRCCS) Humanitas Clinical Institute (protocol number 618/17). All the surgeries were performed by a single surgeon specialized in hip arthroscopy. Symptomatic FAI was defined as anterior hip or groin pain resistant to conservative therapies for at least six months, with positive provocation maneuvers (flexion, adduction, internal rotation) on physical examination and corresponding radiologic findings. Prior to arthroscopic intervention, all patients underwent a standard anteroposterior pelvis and a modified Dunn view hip radiographs, an MRI arthrogram, and a diagnostic intra-articular injection to confirm that symptoms were attributable to the noted intra-articular pathology [13]. Inclusion criteria encompassed consecutive patients who underwent labral repair and femoral neck reshaping. Patients who underwent labral resection and/or reconstruction, subchondral microfractures, subchondral/intra-articular injections or capsulorrhaphy, and patients with an intraoperative chondral damage Outerbridge grade >2 were excluded. All the patients were positioned supine on a traction table (Advanced Supine Hip Positioning System; Sesto San Giovanni, Milan: Smith+Nephew) using a padded perineal post to avoid pudendal nerve and genital injuries. Access to the hip joint was obtained through the anterolateral, distal anterolateral, and mid-anterior arthroscopic portals; a 70° arthroscope (Smith+Nephew) was used. For each patient, labral tears were repaired using an appropriate number of resorbable all-suture anchors; the femoral neck was reshaped under fluoroscopic guidance with a high-speed burr; and the capsule was closed with non-absorbable sutures.

The included subjects were divided into three groups based on the timing and staging of their bilateral procedures. The two-stage group (TS) included patients who underwent staged bilateral hip arthroscopy with an interval of ≥6 weeks between the procedures, the early-stage group (ES) encompassed patients who underwent the second hip arthroscopy the day after the first procedure, and the single-stage group (SS) included patients who underwent simultaneous bilateral hip arthroscopy under a single anesthetic. Assignment to treatment groups was not randomized. Group selection was based on shared decision-making between the patient and surgical team, taking into account patient preference, rehabilitation goals, and logistical considerations. This real-world approach reflects typical clinical decision-making but introduces potential for selection bias. To reduce confounding due to non-random group assignment, propensity score matching was employed. Propensity scores were estimated using logistic regression, incorporating age, gender, and body mass index (BMI) as covariates. A matching for gender, age, and BMI was done using a 2:1:1 ratio. Matching was conducted using the MatchIt package in R software (Vienna, Austria: R Foundation for Statistical Computing) within the RStudio environment (Posit Software; Boston, MA: Posit PBC). Standardized mean differences (SMDs) were assessed postmatching, with SMDs <0.1 indicating adequate balance between groups. Patient-reported outcome measures (PROMs) were collected preoperatively and two years after the last procedure for both hips. The following validated instruments were used to assess clinical outcomes: Hip Outcome Score - Activities of Daily Life (HOS-ADL) [14], Hip Outcome Score - Sports Subscale (HOS-Sport) [14], International Hip Outcome Tool-12 (iHOT-12) [15], and visual analog scale (VAS) for pain [16]. Preoperative scores were recorded within one month before the first surgical procedure. Any major complication (nerve injury, deep vein thrombosis, infection) was recorded. Postoperative scores were collected at the two-year follow-up visit. Patients reported outcomes for each side independently, with appropriate separation of left and right hip scores. Return-to-sport rate, patient satisfaction on a numerical rating scale from 1 to 10, and complication rates were also documented as secondary outcomes. All patients, regardless of group (SS, ES, or TS), followed the same standardized rehabilitation pathway at our institution. Immediate postoperative partial weight-bearing was allowed for all patients and maintained for one month. Physical therapy began on postoperative day 0, with a focus on assisted ambulation and early range-of-motion exercises. During the first month, patients performed isometric strengthening and used continuous passive motion to maintain controlled hip mobility and reduce stiffness. No braces were used in our protocol. Patients were discharged from the hospital after two postoperative nights, in accordance with institutional protocol, unless complications or logistical issues arose.

Statistical analysis of patient-reported outcome measures was performed to compare groups. Data distribution was assessed using the Shapiro-Wilk test to determine normality. For normally distributed variables, group comparisons were conducted using analysis of variance (ANOVA), while non-normally distributed data were analyzed using the Kruskal-Wallis H test. Results are presented as means with standard deviations or medians with interquartile ranges, as appropriate. Effect sizes were calculated to assess the magnitude of differences, and a p-value of less than 0.05 was considered statistically significant.

## Results

Patient characteristics

Three groups were considered as follows: the two-stage (TS) group with 60 patients, the early-stage (ES) group with 30 patients, and the single-stage (SS) group with 30 patients. In the TS group, there were 56 males (93.3%) and four females (6.7%); and in the ES and SS group, there were 28 males (93.3%) and two females (6.7%). Mean age was 32.8±8.6 for the TS group, 31.3±7.8 for the ES group, and 31.1±6.9 for the SS group. Mean BMI was 24.2±2.3 for the TS group, 24.9±1.5 for the ES group, and 25.9±3.1 for the SS group. Baseline demographic and perioperative characteristics are summarized in Table 1.

**Table 1:** Patient characteristics and perioperative data of the study population, stratified by surgical strategy (two stage, early stage, and single stage). TS: two stage; ES: early stage; SS: single stage

Groups	TS	ES	SS
Patients	60	30	30
Males	56	28	28
Females	4	2	2
Mean age (years)	32.8±8.6	31.3±7.8	31.1±6.9
Mean BMI	24.2±2.3	24.9±1.5	25.9±3.1
Surgical time (min)	173.4±25.5	176.2±23.9	168.9±53.3
Total length of stay (nights)	4.2±0.7	3.2±0.4	2.2±0.3
Postoperative length of stay (nights)	2.1±0.4	2.1±0.3	2.2±0.4

Surgical time, length of stay, and complication rates

Mean cumulative surgical time was 173.4±25.5 min in the TS group, 176.2±23.9 min in the ES group, and 168.9±53.0 min in the SS group. There was no significant difference in surgical times among the three groups (p=0.663038). No major complications were recorded. Total length of stay was significantly decreased in the SS group (p<0.00001). Mean cumulative length of stay was 4.2±0.7 nights for the TS group, 3.2±0.4 for the ES group, and 2.2±0.3 for the SS group. No significant difference in postoperative length of stay was observed between groups (p=0.556838). Mean postoperative length of stay was 2.1±0.4 for the first hip and 2.1±0.3 for the second hip in the TS group, 2.2±0.4 for the ES group, and 2.2±0.4 for the SS group. None of the patients required a prolonged hospital stay because of postoperative complications. At the two-year follow-up visit, no revisions or conversions to total hip replacement were recorded.

Clinical outcomes

All analyzed scores were significantly improved (p<0.01) at the last follow-up. The three groups showed a reduction in VAS from 6.5±2.6 to 3.4±2.9 for the TS group, from 7.4±2.2 to 2.9±2.8 for the ES group, and from 6.8±2.9 to 3.1±2.3 for the SS group. HOS-ADL increased from 64.1±19.1 to 81.0±9.2 for the TS group, from 59.1±19.0 to 80.5±12.2 for the ES group, and from 60.1±18.2 to 80.4±8.2 for the SS group. HOS-Sport showed an improvement from 51.1±22.2 to 70.4±18.9 for the TS group, from 46.9±26.3 to 69.3±19.1 for the ES group, and from 51.6±20.0 to 68.5±13.3 for the SS group. iHOT-12 increased from 47.8±26.1 to 68.5±26.0 for the TS group, from 36.9±28.3 to 69.3±26.9 for the ES group and from 43.6±31.9 to 65.3±24.9 for the SS group. Return to sport was 78.3% for the TS group, 73.3% for the ES group, and 76.7% for the SS group. The final satisfaction score was 7.8±2.0 for the ES group, 8.3±1.9 for the TS group, and 7.6±2.1 for the SS group. No significant differences in HOS (p=0.940855), HOS-Sport subscale (p=0.893066), iHOT-12 (p=0.707804), VAS (p=0.610847), and satisfaction score (p=0.255933) were found between the three groups. Clinical outcomes are reported in Table 2.

**Table 2:** Clinical and functional outcomes at baseline and final follow-up in the two-stage, early-stage, and single-stage groups. TS: two stage; ES: early stage; SS: single stage; HOS-ADL: Hip Outcome Score - Activities of Daily Life; HOS-Sport: Hip Outcome Score - Sports Subscale; iHOT-12: International Hip Outcome Tool-12; VAS: visual analog scale

Groups	TS	ES	SS
VAS (mean)			
Pre	6.5±2.6	7.4±2.2	6.8±2.9
Post	3.4±2.9	2.9±2.8	3.1±2.3
HOS-ADL (mean)			
Pre	64.1±19.1	59.1±19.0	60.1±18.2
Post	81.0±9.2	80.5±12.2	80.4±8.2
HOS-Sport (mean)			
Pre	51.1±22.2	46.9±26.3	51.6±20.0
Post	70.4±18.9	69.3±19.1	68.5±13.3
iHOT-12 (mean)			
Pre	47.8±26.1	36.9±28.3	43.6±31.9
Post	68.5±26.0	69.3±26.9	65.3±24.9
Return to sport (%)	78.3	73.3	76.7
Satisfaction score (mean)	8.3±1.9	7.8±2.0	7.6±2.1

## Discussion

This study represents the evolution of a single-center approach to bilateral hip arthroscopy for femoroacetabular impingement (FAI), detailing a shift in surgical strategy over time, dictated by a progression in the surgical team's capability and confidence over the study period. The findings from this study suggest that there is no inferiority in clinical outcomes when employing a simultaneous bilateral hip arthroscopy approach compared to early-staged or two-stage bilateral procedures for femoroacetabular impingement (FAI). Patient-reported outcome measures, including HOS - Activities of Daily Living subscale, HOS - Sport subscale, iHOT-12, and VAS, all showed a significant improvement at the two-year follow-up across all groups, with no significant differences in postoperative scores between them. Furthermore, the study reported no major complications, revisions, or conversions to hip replacement in any group.

A crucial insight from this work is the evolution of our center's approach, which is relevant to the query about less experienced surgeons and the learning curve in hip arthroscopy [11]. As surgeons' experience progressed along the learning curve, a decrease in surgical and traction times, complication rates, and reoperation rates was described in the literature [12,17]. Initially, only staged procedures were performed at our center due to surgical experience and institutional protocols, as follows: an early-staged approach was introduced to provide a more balanced strategy, allowing surgeons to manage technical challenges and reduce complications while gaining proficiency. Although this strategy still involved two surgeries and anesthetic exposures, it could reduce some risks, such as prolonged anesthesia and traction times associated with simultaneous procedures [18,19]. As surgeon confidence and intraoperative protocols improved, simultaneous single-stage procedures were introduced. Indeed, a decrease in mean surgical times was observed over time (i.e., early-stage vs. single-stage); however, this reduction did not reach statistical significance, suggesting that comparisons of outcomes between groups remained unbiased despite the temporal improvements in operative efficiency. Accordingly, Matsuda et al. described simultaneous bilateral hip arthroscopy as a viable option for selected patients and experienced surgical teams, facilitating expedited recovery and potential cost savings due to a single surgery and rehabilitation period [20]. This implies that staged procedures, including the early-staged approach, could be a suitable and safer option for less experienced surgeons, since staged procedures may reduce some risks associated with simultaneous procedures. These findings are supported by other available literature. A systematic review by Fernandez et al. concluded that bilateral hip arthroscopy (whether simultaneous or staged) exhibits similar efficacy and safety when compared with unilateral hip arthroscopy, with no significant differences in patient-reported outcomes, return to sport, traction time, or complications [9]. Importantly, this review also explicitly states that simultaneous procedures may be limited to more experienced hip arthroscopists who are able to perform them more efficiently, due to the increased traction and anesthesia time [9]. In our study, no major complications were observed in the three groups at the two-year follow-up visit. Unfortunately, this study's retrospective approach may underestimate minor postoperative complications, such as pain, local hematomas, edema, temporary nerve palsy, and perineal numbness. However, none of the patients required a prolonged hospital stay because of postoperative complications.

In this study, comparable clinical outcome scores and return to sport rates were observed between simultaneous and staged approaches for bilateral hip arthroscopy. Plus, no significant differences were observed in satisfaction rates between the two-stage and early-stage/single-stage. This reflects the real-world approach of this study - group selection was based on shared decision-making between the patient and surgical team, taking into account patient preferences, rehabilitation goals, and logistical considerations. These results are largely consistent with findings from other sources. In particular, Mei-Dan et al. conclude that simultaneous FAI surgery does not lead to higher rates of complications, postoperative pain, analgesic use, or side effects, and that return to daily activities and clinical outcome scores are similar to staged procedures with the advantage of a single rehabilitation period [21]. Degen et al. further reinforce this, showing that simultaneous bilateral hip arthroscopy is safe and effective, resulting in similar improvements in patient-reported outcomes at one-year follow-up compared to staged bilateral procedures, with comparable complication rates [22]. Foo et al. similarly found that patients undergoing simultaneous bilateral arthroscopy for FAI achieved comparable minimum two-year follow-up outcomes when compared with staged and unilateral arthroscopy [23]. They also reported no differences between groups in revision or arthroplasty conversion rates when adjusted for follow-up time. Among the benefits of simultaneous bilateral hip arthroscopy, these studies reported limited exposure to anesthesia compared with staged approaches, shorter overall recovery time, and a single rehabilitation course for both hips [20-22]. Moreover, simultaneous procedures are considered cost-effective due to reduced hospitalizations and consolidated operative and facility fees [21-23]. Consistent with these findings, our study demonstrated that the single-stage group had a significantly shorter mean total length of hospital stay compared to staged groups, underscoring an important advantage in reducing inpatient time. This not only benefits patients by minimizing hospital-associated risks, such as infections and improving overall satisfaction, but also reduces healthcare resource utilization, which may translate into meaningful cost savings for healthcare systems [24,25].

This study has several strengths. First, patient selection was clearly defined, ensuring a homogeneous cohort of individuals undergoing bilateral hip arthroscopy for symptomatic FAI. Second, the structured grouping of patients into simultaneous, early-staged, and two-stage procedures allowed for a meaningful comparison of timing strategies within a consistent clinical framework. Third, the use of standardized and validated patient-reported outcome measures (PROMs) (HOS-ADL, HOS-Sport, iHOT-12, and VAS) at baseline and at two-year follow-up provides reliable and comparable outcome measures. Additionally, the application of propensity score matching minimized demographic variability between groups, thereby strengthening the internal validity of the comparative analyses.

Like many retrospective cohort studies in surgical outcomes, this study presents several potential biases and limitations that are important to acknowledge for a comprehensive understanding of findings. Despite the use of propensity score matching, unmeasured confounders may still be present, and the retrospective design limits control over missing data. Follow-up assessments were not blinded, which may introduce observer or reporting bias. Group selection was based on a shared decision-making process involving the patient and the surgical team, considering factors, such as patient preference, rehabilitation goals, and logistical considerations, and this approach, while reflective of real-world clinical practice, inherently introduces selection bias as follows: patients who chose or were directed to simultaneous surgery might have been more motivated or physically fit, potentially influencing their outcomes irrespective of the surgical approach. Because the early-stage procedures performed at a lower level of overall surgical experience within the center compared to the later introduced simultaneous procedures, this evolution in technique and proficiency over the study's timeframe could influence outcomes, leading to temporal bias. Thus, a single surgeon's experience ensures consistency in surgical technique within the study but inherently limits the generalizability of the results to other surgeons or institutions, particularly those with different levels of experience or surgical volume. Additionally, our cohort was male predominant, reflecting the demographic profile of patients treated at our institution and consistent with epidemiological studies, but this may limit the generalizability of our findings to a more gender-balanced population [26]. Finally, the absence of long-term outcomes restricts our ability to evaluate the durability of the results beyond the two-year follow-up period.

## Conclusions

Our study demonstrated that there is no inferiority in clinical outcomes when using a simultaneous approach compared to more staged approaches, and the single-stage group had a significantly shorter mean total length of hospital stay compared to staged groups, underscoring an important advantage in reducing inpatient time. This not only benefits patients by minimizing hospital-associated risks, such as infections and improving overall satisfaction, but also reduces healthcare resource utilization, which may translate into meaningful cost savings for healthcare systems. However, more comprehensive clinical characterization and expanded outcome reporting, including long-term follow-up and objective functional assessments, are needed to strengthen the evidence base and confirm the durability and generalizability of these findings.
